# Community-acquired Methicillin-resistant *Staphylococcus aureus*, Uruguay

**DOI:** 10.3201/eid1106.041059

**Published:** 2005-06

**Authors:** Xiao Xue Ma, Antonio Galiana, Walter Pedreira, Martin Mowszowicz, Inés Christophersen, Silvia Machiavello, Liliana Lope, Sara Benaderet, Fernanda Buela, Walter Vicentino, María Albini, Olivier Bertaux, Irene Constenla, Homero Bagnulo, Luis Llosa, Teruyo Ito, Keiichi Hiramatsu

**Affiliations:** * Juntendo University, Tokyo, Japan;; † Hospital Maciel, Montevideo, Uruguay;; ‡ Centro de Asistencia del Sindicato Médico del Uruguay, Montevideo, Uruguay;; § Ministerio del Interior, Montevideo, Uruguay;; ¶ Ministry of Public Health, Montevideo, Uruguay

**Keywords:** molecular epidemiology, bacteremia, Antibiotic resistance, streptococcus pneumoniae, pneumococcal, vaccine, children

## Abstract

A novel, methicillin-resistant *Staphylococcus aureus* clone (Uruguay clone) with a non–multidrug-resistant phenotype caused a large outbreak, including 7 deaths, in Montevideo, Uruguay. The clone was distinct from the highly virulent community clone represented by strain MW2, although both clones carried Panton-Valentine leukocidin gene and *cna* gene.

Since the 1990s, methicillin-resistant *Staphylococcus aureus* (MRSA) infections have been increasingly recognized in the community, and MRSA strains isolated from patients with community-associated cases have been called community-associated MRSA (CA-MRSA) (1). CA-MRSA strains have been reported to differ from isolates from hospitals (healthcare-associated MRSA; HA-MRSA) in many characteristics such as susceptibility to antimicrobial drugs, types of staphylococcal cassette chromosome (SCC) *mec* element, and repertoires of exotoxin gene. In Uruguay, MRSA strains are among the most prevalent nosocomial pathogens. In late 2001, we observed a case in a young man with recurrent boils who visited an outpatient clinic. An MRSA strain that was susceptible to other drugs was isolated from the patient. After that, pediatric infections associated with similar strains were observed (2). The initial sporadic cases were followed by an epidemic increase of infections in the community, hospitals, and jails. We began to record the microbiologic data and analyze cases together with the National Antimicrobial Resistance Surveillance Network belonging to the Public Health Ministry in Uruguay, and we concluded that a large outbreak of CA-MRSA strains occurred in Uruguay. Here we report the emergence of a novel CA-MRSA clone, which has been shown by multilocus sequence typing (MLST) and SCC*mec* type to be distinct from the midwestern CA-MRSA strain.

## The Study

We studied patients with non–multidrug-resistant MRSA infections identified at 2 hospital centers in the metropolitan area of Montevideo, Uruguay, Hospital Maciel and Centro de Asistencia del Sindicato Médico del Uruguay, from January 2002 to October 30, 2003. A total of 125 *S*. *aureus* strains that were resistant to oxacillin alone or to erythromycin in addition were isolated from outpatients and inpatients. Since 1 of our members noticed some cases of pyogenic infections in a prison, we conducted a sentinel study of skin and soft tissue infection (SSTI) in the 2 main prisons from May to June in 2003. We isolated 40 non–multidrug-resistant MRSA strains from 58 inmates with SSTIs. Of these 40 strains, 17 were randomly selected to be analyzed. Susceptibilities to 8 antimicrobial drugs (oxacillin, vancomycin, gentamicin, rifampin, ciprofloxacin, erythromycin, clindamycin, and trimethoprim-sulfamethoxazole) were tested by the Kirby Bauer disk diffusion test (Becton Dickinson, Cockeysville, MD, USA). Production of PBP2´ and protein A were verified by MRSA Screen latex PBP2´ (Denka Seiken-Oxoid Ltd, London, UK) and latex slide agglutination kits (Oxoid, Hampshire, UK), respectively. Most (133/142, 94%) showed heterogeneity in the degree of resistance to oxacillin, since double halos or haze zones were observed around the disk containing 1 μg of oxacillin.

**Figure 1:**
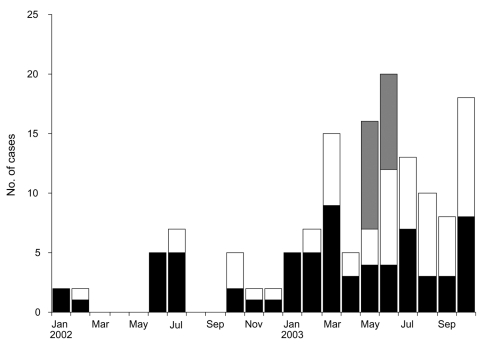
The monthly accumulation of cases of infections due to non–multidrug-resistant MRSA strains from January 2002 to October 2003. Black blocks indicate numbers of strains that were isolated from patients in the public hospital (Hospital Maciel), white indicates strains from a private hospital (Centro de Asistencia del Sindicato Médico del Uruguay), and gray indicates strains from 2 prisons (Libertad and Comcar).

The course of the outbreak during the 22 months is shown in Figure 1. The number of MRSA infections increased greatly in 2003. The Table summarizes the cases in which non–multidrug-resistant MRSA strains were isolated. Of the 125 case-patients, 112 were adults. The mean age was 39.7 years, which was lower than that of case-patients infected with HA-MRSA strains (mean age 59 years) reported previously (3). We classified the cases as community-associated if MRSA was isolated from cultures performed within 48 hours after admission to hospitals and excluded patients who had previously noted criteria for risk factors of HA-MRSA acquisition: recent hospitalization (within the last 6 months); use of medical devices (such as a permanent indwelling catheter or percutaneous medical device); exposure to healthcare services, including invasive or surgical procedures; residence in a long-term care facility; and any known antimicrobial drug use within the past year (4,5). Community-associated cases were dominant (78%). The predominant infection type in adults was skin and soft tissue infection (n = 86) such as abscesses, boils, and cellulitis, followed by respiratory tract infections, among which 12 of 14 were pneumonia. Four of 14 adult patients with respiratory tract infections exhibited symptoms of acute severe pneumonia, with histopathologic findings of "necrotizing pneumonia," and all died after bacteremia developed. Besides developing in these 4 patients with fatal cases, bacteremia developed in 9 other patients, and 3 of them died. The sites of infection preceding the bacteremia for these 3 patients were skin and soft tissue, bone and joint (septic arthritis), and unknown (classified as septic syndrome in Table), respectively. Bacteremia also developed in 3 pediatric patients. In total, bacteremia developed in 17 patients, and 7 died during the study period. We studied the molecular microbiologic characteristics of 68 isolates: 16 from the patients in whom bacteremia developed, 35 randomly selected from all case-patients, and 17 from the inmates.

**Table:** Clinical presentation of 125 MRSA-infected case-patients, Montevideo, Uruguay*

Clinical feature	Adult patients infected in†			Pediatric patients infected in‡	
	Community	Hospital	Unknown	Community	Hospital
Skin and soft tissue					
Abscess	26 (4) ¶			3 (3)	
Boils	20 (5)			1(1)	
Cellulitis	15 (3)	2 (2) ¶		2 (1)¶	
Hidradenitis	3 (2)				
Myositis	1 (1)				
Wound infection	8 (3)	11 (3)			
Infected atopic dermatitis					1 (1)¶
Respiratory tract					
Upper respiratory tract infection				4 (1)	
Necrotizing pneumonia	4 (3)¶#				
Pneumonia	1 (1)	3 (1) ¶			
Ventilator-associated pneumonia§		4(4)			
Colonization in respiratory tract		2(2)			
Catheter-associated infection					1 (1) ¶
Cerebrospinal fluid shunt					1(1)
Bone and joint infection	2 (1)¶	1 (1)¶			
"Sepsis" syndrome	5 (4) ¶		4 (2)		
Total	85 (27)	23 (13)	4 (2)	10 (6)	3 (3)

*MRSA, methicillin-resistant Staphylococcus aureus; parenthesis indicate the numbers of case-patients whose MRSA isolates were analyzed in this study. †The range and mean age were 16–82 years and 39.7 years, respectively. The number of male and female case-patients were 65 (58%) and 47 (42%), respectively. Twenty-nine case-patients required hospitalization. ‡The range and mean age were 16–82 years and 6 years, respectively. The numbers of male and female case-patients were 8 and 5, respectively. One patient required hospitalization. §Ventilator-associated pneumonia of the patients in an intensive care unit. ¶Besides 9 cases of sepsis syndrome, some of the other case categories were also bacteremic. They were 1 abscess, 4 necrotizing pneumonia, 2 bone and joint, 2 cellulitis, 1 infected atopic dermatitis, and 1 catheter-associated infection. #A strain isolated from 1 of the patients was lost for analysis in this study.

Pulsotypes, coagulase isotypes, SCC*mec* types, and exotoxin gene repertoires were examined by the methods indicated in the footnotes of the Table A1. Among 6 pulsotypes identified in the Uruguay strains, 38 (74.5%) of 51 isolates from patients and all 17 isolates from the inmates, had related pulsotypes within 4 band differences designated A1–A4 (Table A1). All of them produced type-4 coagulase and carried type IVc SCC*mec* element, 53 of 55 carried *lukS*,*F*-*PV* genes, and 51 of 55 carried the *cna* gene. Isolates from 11 of 16 bacteremic case-patients, including 6 who died, belonged to pulsotype A. Evidence shows that a clone (pulsotype A-SCC*mec* IVc), which possessed both *cna* and *lukS*,*F*-*PV* genes, caused the outbreak in Uruguay.

Other pulsotype strains carried primarily other SCC*mec* elements, such as type-II, type-IVa, or type-V, and produced type-2, -5, or -7 coagulase and did not carry *lukS*,*F*-*PV* genes. Notably, 49 (96%) of 51 strains isolated from both community-associated and healthcare-associated cases carried either type-IV or type-V SCC*mec* element, which have been found in CA-MRSA strains (6–8).

**Figure 2:**
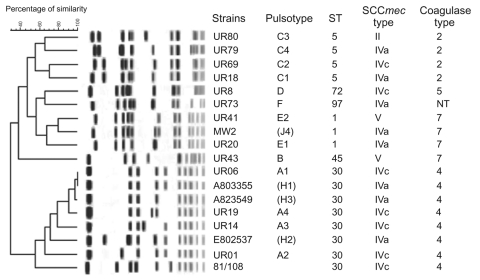
Dendrogram of pulsed-field gel electrophoresis (PFGE) banding pattern of representative Uruguay clone. Pulsotypes of representative Uruguay strains, a CA-MRSA strain isolated in the United States (MW2), 3 CA-MRSA strains isolated in Australia (A803355, A823549, and E802537), and a Japanese strain isolated from an outpatient (81/108) were compared by using a BioNumerics software program (Applied Maths, Sint-Martens-Latem, Belgium). Similarity coefficient was calculated by using Pearson correlation with position tolerance of 5%, and cluster analysis was performed by the unweighted pair-group method. Pulsotypes in parentheses indicate the types previously reported (7). PFGE was performed for 22 h with a CHEF MAPPER (Bio-Rad, Hercules, CA, USA) with a pulse time of 5 s to 40 s. *Sma*I-restriction patterns of the tested strains and reference strains were compared by using BioNumerics software. Genotypes of representative strains were determined by multilocus sequence typing as described by Enright et al. (9). Sequence type (ST) and clonal complex were assigned using programs in the *S. aureus* multilocus sequence typing database (http://www.mlst.net).

We compared characteristics of outbreak strains with those of previously investigated CA-MRSA strains isolated in the United States (MW2) and Australia (A803355, A823549, and E802537), and a strain isolated from an outpatient in Japan in 1981 (81/108) (Figure 2). All of them possessed both *cna* and *lukS*,*F*-*PV* genes as well. The dominant outbreak strains belonged to ST-30, which was the same as Australian and Japanese strains and distinct from MW2 (ST-1). In addition, we found that the pulsotype of strain A803355 (reported previously as H1) was identical to pulsotype A1, the most representative pulsotype among tested strains. Pulsotypes of other 2 Australian strains, A823549 and E8025347, were classified into the same cluster as A, while that of 81/108 showed a similar type on pulsed-field gel electrophoresis (PFGE) to pulsotype A. In contrast, only 2 Uruguay isolates,UR20 and UR41, had a PFGE pattern similar to that of MW2.

## Conclusions

The outbreak of CA-MRSA in Uruguay involved >1,000 patients and ≤12 deaths, when the data after this study period are added. According to a follow-up survey conducted at jails from May to October 2003, 890 of 1,142 inmates were infected with similar pyogenic infections after an outbreak of scabies (10). Five patients required hospitalization. Boils and abscesses in the buttocks and neck were the most prevalent infections (85%), followed by hidradenitis and cellulitis. The prevalence of a new clone represented by UR 6 (Uruguay clones) is considered to have been the cause of this large outbreak. We are conducting a further study of isolates from patients after this study period and isolates from inmates in a follow-up survey, and a final conclusion awaits those results.

We have suggested 2 genes as important candidates for high virulence in the midwestern CA-MRSA strains represented by strain MW2 (11), which is distributed in the United States and Europe (12,13). Since the Uruguay clone shared *lukS*,*F*-*PV* genes and *cna* gene with the midwestern CA-MRSA clone, this study strengthened the likelihood that these 2 genes are contributors for high virulence. Their genotypes, however, were completely different, and we now appear to have 2 distinct clones of highly virulent CA-MRSA. That certain CA-MRSA strains, identified in Australia as carrying the *luk*-*PV* and *cna* genes, had an identical PFGE pattern with UR6 does not imply that the same CA-MRSA clone has been disseminated between Uruguay and Australia because these 2 clones had distinct SCC*mec* elements, IVc and IVa, respectively. Since the MRSA clone originated when the SCC*mec* was integrated into the chromosome of a *S*. *aureus* strain, the 2 MRSA clones are understood to have originated independently by acquiring different SCC*mec* genes in their respective countries (14,15). In this regard, the difference in the SCC*mec* type was not reflected in the PFGE pattern. This example provides an excellent illustration of the fact that clonality cannot be judged on PFGE pattern alone.

Nonetheless, CA-MRSA strains with identical PFGE and MLST patterns possessing *lukS*,*F*-*PV* genes and *cna* genes exist in both Uruguay and Australia. This finding may indicate the existence of a genetically stable, virulent, multidrug-susceptible *S*. *aureus* (MSSA) clone in a community that extends beyond country borders and across the ocean. The MSSA clone, which has *lukS*,*F*-*PV* and *cna* genes, is established in the community and occasionally acquires SCC*mec* when the use of β-lactam antimicrobial drugs is increased to a stressful level for the survival of the MSSA clone in the community. Since we have also found MRSA strains isolated in the 1980s in Japan with a similar genotype to that of UR6 and possessing *PVL* and *cna* genes, the outbreak observed in Uruguay could occur in any part of the world if social or medical predisposing conditions are met.

## Appendix

**Table A1:** Characteristics of 51 MRSA strains isolated in the study period and 17 MRSA strains isolated in prisons*

Pulsotype	SCC*mec* type†	No. of isolates	Coagulase-isotype‡	Exotoxine genes§					
				PVL	CNA	SEH	TSST1	bac	Clinical category¶
MRSA strain isolated from patients									
A1	IVc	28	4	+	+	–	–	–	C-abscess(A-3[died, 1#], P-1), C-boils(A-5, P-1) C-cellulitis (A-1, P-1#) H-cellulitis(A-2[1#]), H-wound infection(A-3) C-hidradenitis (A-2) C-myositis (A-1) C-necro pneumo(A-2[died, 2#]), H-VAP(A-2) H-bone joint (A-1[died, 1#]), C-sepsis (A- 3[died, 1#])
A1	IVc	1	4	+	–	–	–	–	C-wound infection (A-1)
A1	IVc	1	4	–	–	–	–	–	H-VAP(A-1)
A1	IVc	1	4	–	+	–	–	–	C-sepsis(A-1)#¶
A2	IVc	5	4	+	+	–	–	–	C-abscess (A-1, P-2) C-cellulitis (A- 1) C-bone joint(A- 1)
A3	IVc	1	4	+	+	–	–	–	C- necro pneumo (A-1[died, 1] #)
A4	IVc	1	4	+	+	–	–	–	C-cellulitis (A-1)
B	V	1	7	–	+	–	+	–	H-atopic dermatitis (P-1)#¶
C1	IVa	2	2	–	–	–	-	–	H-catheter-associated infection(P-1)# H-cer spin fluid shunt (P-1)
C2	IVc	1	2	–	–	–	+	–	C-wound infection(A-1)
C2	V	1	2	–	+	–	+	–	C-wound infection (A-1)
C3	II	2	2	–	–	–	–	–	H-VAP (A-1), U-sepsis syndrome (A-1)#¶
C4	IVa	1	2	–	–	–	+	–	U-sepsis syndrome (A-1)#
D	IVc	1	5	–	–	–	–	–	C-up resp tract (P-1)
E1	IVa	1	7	–	+	+	–	+	C-pneumonia (A-1)
E2	V	1	7	–	+	+	–	+	H-pneumonia (A-1)#
F	IVa	2	NT	–	–	–	–	–	H-respiratory tract colonization (A-2)
MRSA strains isolated from prison									
A1	IVc	13	4	+	+	–	–	–	C-cellulitis (A-11), C-hidradenitis(A-2)
Reference strains									
G	III		4	–	+	–	–	+	H-VAP(A-1)
(J4)	IVa		7	+	+	+	–	+	C-necro pneumo (died)

*MRSA, methicillin-resistant *Staphylococcus aureus*; hidradenitis, hidradenitis suppurativa; necro pneumo, necrotizing pneumonia; bone joint, bone and joint infection; VAP, ventilator-associated pneumonia; up resp tract, upper respiratory tract infection. †SCC*mec* type was determined by polymerase chain reaction (PCR) amplification using the primer's sets described previously (6–8). The primers used for type IVc SCC*mec* element, 4c1 (5´-TCTATTCAATCGTTCTCGTATTT-3´) and 4c2 (5´-TCGTTGTCATTTAATTCTGAACT-3´), were designed based on the nucleotide sequence of type IVc SCC*mec* element of strain 81/108, deposited in DDBJ/EMBL/GenBank databases accession no. AB096217. ‡Coagulase isotyping: the type of coagulase was determined by coagulation inhibition test using commercially available neutralizing antisera specific to each of the 8 coagulase types I to VIII, according to the method recommended by manufacturer (Denka Seiken Co. Ltd, Niigata, Japan). §Exotoxin genes were identified by PCR with sets of primers as follows:*PVL* (Panton-Valentine leukocidin), *PVL-F* (5´-ATGTCTGGACATGATCCAA-3´) and *PVL-R* (5´-AACTATCTCTGCCATATGGT-3´); CNA (collagen-binding protein), *cna1* (5´-ACACCAGACGGTGCAACAATTA-3´) and *cna2* (5´-AGCAATACCGTTTGCATCTGTTA-3´); *SEH* (staphylococcal enterotoxin H ), entH-F (5´-ATTCACATCATATGCGAAAGCAG-3´), and *entH-R* (5´-ATGTCGAATGAGTAATCTCTAG-3´); *TSST1* (toxic shock syndrome toxin-1), *TSST1A* (5´-TGATATGTGGATCCGTCAT-3´), and *TSST1B* (5´-AAACACAGATGGCAGCAT-3´); *bac* (bacteriocine gene found in MW2), *epiA-F* (5´-GACGAACGTATTACAAGTCATA-3´), and *epiB-R* (5´-TAAGTACGCTGCTTCAGATATA-3´). ¶Clinical categories are based on the Table . Those of the strains not listed in the Table are in italic Onset: onset of infection in either community(C), hospital (H), or unknown (U). Numbers in parenthesis indicate the number of cases: A, adult cases; P, pediatric cases. The number of cases in which patient died is indicated in brackets. #Patient had bacteremia.
