# Correction, vol. 11, no. 6

**DOI:** 10.3201/eid1108.C11108

**Published:** 2005-08

**Keywords:** errata, erratum, correction

In “Community-acquired Methicillin-resistant *Staphylococcus aureus*, Uruguay” by Xiao Xue Ma et al., errors occurred on pages 973 and 974.

The first sentence of the abstract should read as follows: A novel, methicillin-resistant *Staphylococcus aureus *clone (Uruguay clone) with a non–multidrug-resistant phenotype caused a large outbreak, including 7 deaths, in Montevideo, Uruguay.

The first sentence of the article should read as follows: Since the 1990s, methicillin-resistant *Staphylococcus aureus *(MRSA) infections have been increasingly recognized in the community, and MRSA strains isolated from patients with community-associated cases have been called community-associated MRSA (CA-MRSA).

The first sentence of Figure 1 legend (p. 974) should read as follows:

The monthly accumulation of cases of infections due to non–multidrugresistant MRSA strains from January 2002 to October 2003.

The corrected article appears online at http://wwwnc.cdc.gov/eid/article/11/6/04-1059_article.htm.

We regret any confusion these errors may have caused.

